# Heterochely and handedness in the orange mud crab *Scylla olivacea*: implication for future culture practice optimisation

**DOI:** 10.7717/peerj.15143

**Published:** 2023-04-03

**Authors:** Rusydi Rozaimi, Alexander Chong Shu-Chien, Youji Wang, Sutikno Sutikno, Mhd Ikhwanuddin, Xi Shi, Ghazali Azmie, Hanafiah Fazhan, Khor Waiho

**Affiliations:** 1 Higher Institution Centre of Excellence (HICoE), Institute of Tropical Aquaculture and Fisheries, Universiti Malaysia Terengganu, Kuala Nerus, Malaysia; 2 Centre for Chemical Biology, Universiti Sains Malaysia, Penang, Malaysia; 3 School of Biological Sciences, Universiti Sains Malaysia, Penang, Malaysia; 4 International Research Center for Marine Biosciences, Ministry of Science and Technology, Shanghai Ocean University, Shanghai, China; 5 Research Center for Applied Zoology, National Research and Innovation Agency (BRIN), Cibinong-Bogor, Indonesia; 6 STU-UMT Joint Shellfish Research Laboratory, Shantou University, Shantou, China; 7 Engineering Technology Research Center of Henan Province for Aquatic Animal Cultivation, College of Fisheries, Henan Normal University, Xinxiang, China

**Keywords:** Heterochely, Handedness, Portunid, Orange mud crab, *Scylla olivacea*

## Abstract

Asymmetric body traits in bilateral organisms are common and serve a range of different functions. In crustaceans, specifically among brachyuran crabs, heterochely and handedness in some species are known to aid in behavioural responses such as food acquisition, and sexual and territorial displays. However, the heterochely of the intertidal mud crab genus *Scylla* is still poorly understood. This study investigated the cheliped morphometric characteristics of orange mud crab *Scylla olivacea* and the relation of heterochely and handedness to sex. *Scylla olivacea* is heterochelous, with predominant right-handed (70.2%). Three morphometric variables, *i.e.*, propodus length (PL), propodus depth (PD), and propodus width (PW) were significantly larger in the right cheliped and the estimated handedness based on these three variables were consistent with the presence of molariform teeth. The effect of sex had no influence on the occurrence of heterochely or handedness. The frequency of left-handedness increased with size, especially in males. We postulate that handedness reversal, a phenomenon seen in other crab species when the dominant hand is lost, also occurs in *S. olivacea*, thereby resulting in a change in left-handedness frequency. The use of chelipeds by males in mate and territorial defenses might provide an explanation for the higher risk of losing a dominant cheliped and thus, higher left-handedness frequency compared to females. Future behavioural research could shed light on the selective forces that affect the handedness distribution in mud crabs. Knowledge on heterochely and handedness of mud crabs could be useful for future development of less aggressive crab populations by claw reversal and the optimisation of limb autotomy techniques.

## Introduction

Asymmetries are common among living organisms, and they can be seen in a variety of forms. Asymmetry, either in size or shape, is a pattern that regularly arises in evolutionary processes (Palmer, 1996; Spani et al., 2020). A random minor variation of a morphological trait from perfect bilateral symmetry is an example of what is referred to as fluctuating asymmetry at the population level. Analysis of the degree to which the right and left sides of the organism’s bilateral characteristics diverge from one another may be used to estimate the level of body asymmetry that an organism possesses, which can be done by using a population sample (Ottaviano & Scapini, 2010). Often, it results from a difference in how an individual’s left and right sides of their body have developed. This state can be interpreted as a deviation from perfect symmetry on the part of an organism or a component of that organism (Van Valen, 1962; Spani et al., 2020). In particular, many decapod crustaceans (Lezcano et al., 2015; Hamasaki & Dan, 2022), including brachyurans, show asymmetry in the shape and size of their chela (Scalici & Gherardi, 2008). The development of heterochely thus results from this disruption of bilateral symmetry (Graham et al., 2010).

The chela, carpus, and merus are the three major cheliped segments of brachyuran crabs. Two primary parts form a chela; the first is a propodus that extends distally and is known as the pollex. The second component is termed as the dactylus, and together they form an appendage that has a pincher-like claw (Claverie & Smith, 2007). Heterochely, in which the two chelae are distinct from one another in terms of size and shape or between the both of them, is a phenomenon that frequently occurs in the Brachyura (Hartnoll, 1982; Silva & Paula, 2008). In most cases, individuals of both sexes exhibit heterochely (Emlen, 2008; Claverie & Smith, 2010). The two chelae are commonly referred to as major and minor chela due to their size disparity. They are also known as the crusher and the cutter based on their functionality in addition to their difference in size (Spani et al., 2020). The major chela can be found on the right side of most crab species, but a small population of left-handed crabs coexists among the species. The occurrence of left-handedness is thought to be due to the reversal of handedness resulting from the major chela being lost from the right side of the crab (Abby-Kalio & Warner, 1989).

*Scylla olivacea* can be found in large numbers throughout most of mangrove forests and in the intertidal zones of the Malaysia’s coastal zones. It has been documented in many mangrove regions, including the waters of west Malaysia in the states of Perak (Matang Mangrove Forest Reserve, Taiping) and Terengganu (Setiu), as well as the seas of east Malaysia in the states of Sabah (Kota Merudu), and Sarawak (Lundu) (Waiho, Fazhan & Ikhwanuddin, 2016b; Fazhan, Waiho & Ikhwanuddin, 2017a). In addition to Malaysia, *S. olivacea* is also found in Thailand (Moser et al., 2005), Indonesia (Fujaya et al., 2020), Vietnam (Macintosh, Overton & Thu, 2002), Bangladesh (Rouf et al., 2021), India (Viswanathan & Raffi, 2015), Japan (Ogawa et al., 2011), and western Australia (Keenan, Davie & Mann, 1998). All four species of mud crabs, including *S. olivacea*, are economically important species and play critical role in sustaining the livelihood of local coastal communities through mud crab fishery and aquaculture.

Most of the research that have been done on chelipeds of mud crabs are related to limb autotomy and regeneration (Quinitio & Estepa, 2011; De la Cruz-Huervana, Quinitio & Corre, 2019; Fujaya et al., 2020; Gong et al., 2022), a process that affects the physiology and behaviour of these animals (Fazhan et al., 2022), and is the primary focus of mud crab aquaculture sector for growth enhancement and optimisation, as well as soft-shell crab production (Fujaya et al., 2020; Rahman et al., 2020). In addition, there is research on the chelae crushing force at multiple gape sizes in *S. olivacea* conducted by Yap, Lin & Todd (2013). However, there are limited knowledge and information regarding the heterochely and handedness of members of the *Scylla* genus, particularly *S. olivacea*. Therefore, in this study, we examined the relationship of heterochely and handedness of the orange mud crab, *S. olivacea,* based on selected cheliped morphometric characteristics. The potential biological functions of heterochely and handedness of *S. olivacea* were further discussed. Knowledge of the presence of heterochely and handedness in *S. olivacea* serves as an essential foundation for future research on limb autotomy and regeneration of mud crabs.

## Materials & Methods

Mud crabs were sampled from Matang Mangrove Forest Reserve, Perak, Malaysia (4°45′N 100°37E). Standard crab pots with trash fish as baits were deployed during low tide and retrieved on the subsequent low tide. Mud crab fishery is permitted at Matang Mangrove Forest Reserve and supports the livelihood of local communities; thus, no specific license is needed for the capture of mud crabs. Upon capture, mud crabs were identified at the species level based on the morphological characters provided by Keenan, Davie & Mann (1998) and Fazhan et al. (2020). *Scylla olivacea* has distinct orange or red through brown to black coloured chelipeds, rounded frontal lobe spines, reduced outer carpus spines, lack of inner carpus spines, and reduced propodus spines. After species identification, crabs were then sexed according to their abdomen shape and characters; males possess triangular-shaped abdomens, whereas females have darkened globular abdomens in mature individuals and slightly rounded abdomens in immature individuals (Ikhwanuddin et al., 2011; Fazhan et al., 2021). Due to the lack of clear and objective morphological character to discern between immature and mature males, previously determined size at sexual maturity (carapace width, CW_50_) of 87 mm CW for *S. olivacea* from the same population was used in this study (Waiho, Fazhan & Ikhwanuddin, 2016b). In addition, the abdomen looseness of each male was also checked; males were considered as mature if the abdomen was not locked to the sternum when probed using a dissecting needle, whereas only the telson can be flipped open in immature males (Waiho et al., 2016a). Only crabs with no physical signs of abnormalities or disease were used. Crabs with missing one or both chelipeds were measured but not included in heterochely analysis. After data collection, all crabs were either released live to the mangrove, returned to local fishers, or used in other mud crab-related studies.

Morphometric measurements of the carapace, including CW—the distance between the tips of the 9th anterolateral spine, internal CW (ICW)—the distance between the groove of the 8th and 9th anterolateral spine, carapace length (CL)—the distance between the middle of the rostrum (between the eye socket) and the most posterior part of the carapace, and that of both chelipeds, including cheliped dactyl length (DL), propodus length (PL), propodus width (PW), propodus depth (PD), and merus length (ML) were measured to the nearest 0.01 mm using digital vernier caliper (Fazhan et al., 2020). In addition, body weight (BW) was weighed to the nearest 0.1 g using a standard digital weighing scale. Tooth types, either molariform or serratiform type, of both chelipeds of males (*n* = 15) and females (*n* = 15) were also analysed, and the large molar tooth on each cheliped was measured to the nearest 0.01 mm. All mean values are expressed using standard deviation unless stated otherwise.

Data analysis was performed in SPSS Statistic ver. 25. CW was chosen as the reference baseline parameter (independent variable) for regression analyses, as it is a commonly used size measurement standard in portunid crabs, including mud crabs, due to the ease of measurement and the lack of abrupt changes while reaching morphometric maturity (Parvizi et al., 2017; Waiho et al., 2017). Data were checked for normality using the Shapiro–Wilk test. Data of cheliped dimensions, *i.e.,* DL, PL, ML, PD and PW, were not normally distributed (*P* < 0.05), except for DL of left cheliped (*P* = 0.138). As the paired-samples *t*-test is robust to violations of normality (Wiedermann & Von Eye, 2013), paired comparisons of left and right cheliped dimensions were conducted, with any violations of normality being noted. The Chi-square test of independence was conducted to determine the relationship between handedness (left or right) and sex, maturation status, and body size (CW size class); the assumptions of the chi-square test were met, and all expected cell frequencies were above five. Binomial logistic regression was conducted using handedness as the dependent variable and sex, maturation status, CW, ICW, and CL as the predictor variables to predict the probability of *S. olivacea* being left- or right-handed based on the independent variables. Regression analyses were conducted on log-transformed variables; cheliped variables based on handedness and sex were considered as dependent variables, whereas CW was regarded as the independent variable. Owing to the lack of significant difference in handedness according to maturation status, this variable was collapsed in the subsequent regression analysis. However, we maintained sex as an important category throughout regression analysis to aid in understanding the handedness pattern in major and minor chelipeds of *S. olivacea*. Statistical significance level was set at *P* = 0.05.

## Results

**Figure 1:**
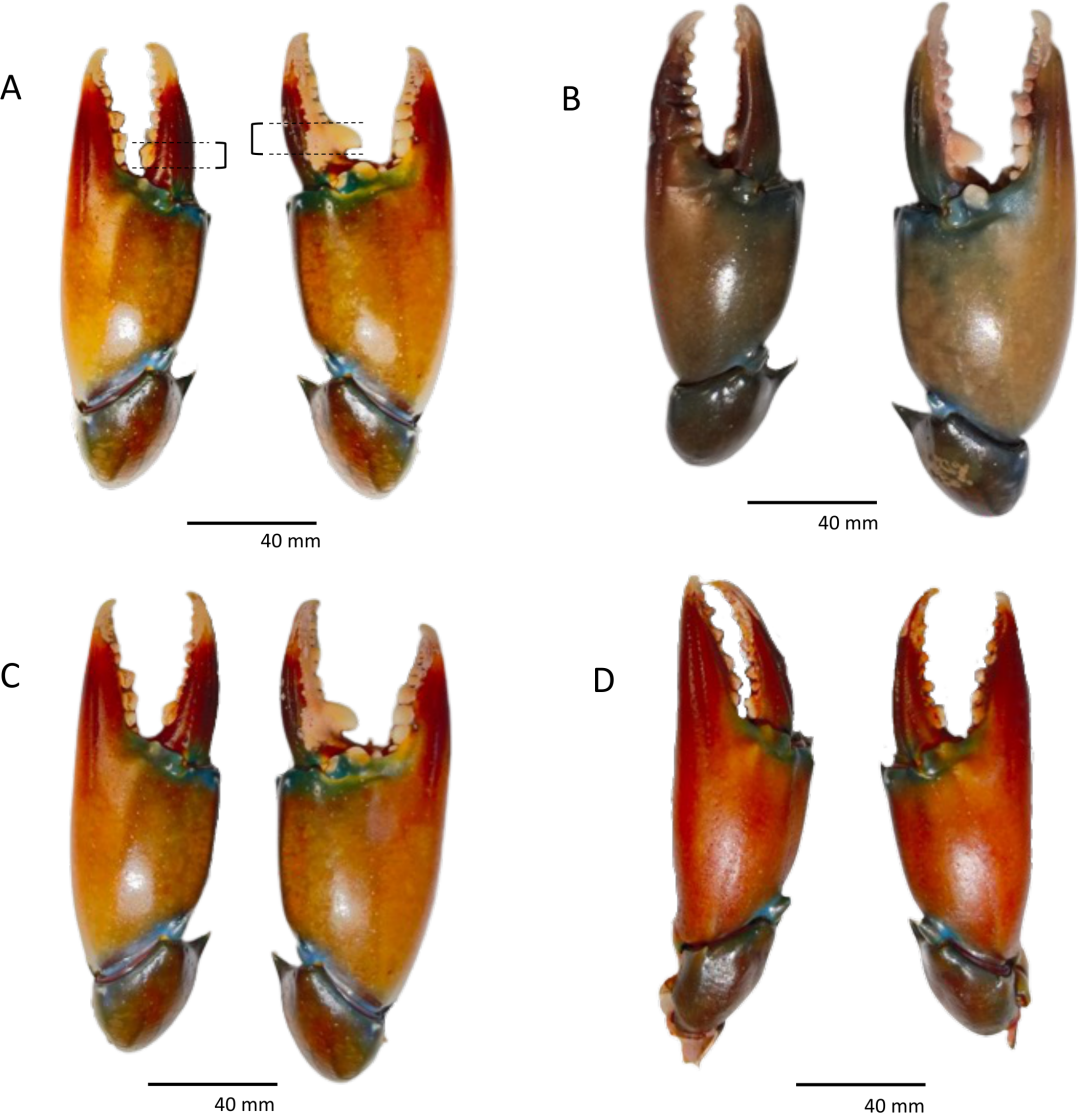
(A–D) Examples of heterochely and the presence of large molariform teeth on major cheliped. The measurement points for molariform teeth are represented by dotted lines as in (A). (A) Left molariform = 4.43 mm, right molariform = 5.48 mm. (B) Left molariform = 4.76 mm, right molariform = 5.56 mm. (C) Left molariform = 3.61 mm, right molariform = 5.93 mm. (D) Left molariform = 4.55 mm, right molariform = 3.14 mm.

A total of 151 individuals were obtained, and all had both chelipeds intact (Table 1). Only two mature males were missing their left cheliped dactylus (Table S1). *Scylla olivacea* is heterochelous, in which PL, PD, and PW of right cheliped were 1.28 ×, 1.39 ×, and 0.74 × larger than that of left cheliped (PL: *t* (150) = 3.827, *P* < 0.001; PD: *t* (150) = 4.63, *P* < 0.001; PW: *t* (150) = 3.744, *P* < 0.001). However, DL and ML were not significantly difference between left and right chelipeds (DL: *t* (148) = 0.343, *P* = 0.732; ML: *t* (150) = 1.442, *P* = 0.151). In addition, it was observed that crabs, in general, had a larger tooth on the major cheliped, and the first major tooth on the right cheliped was 1.18 times larger than that on the left cheliped (*t* (18) = 2.314, *P* = 0.033; the assumption of normality was not violated, as assessed by Shapiro–Wilk’s test, *P* = 0.141 for right tooth and *P* = 0.225 for left tooth). Manual handedness assessment by comparing the PL, PD and PW of both chelipeds revealed that handedness based on these three cheliped morphometric characters was in synced with the handedness estimated *via* the presence of large molariform cheliped on the major cheliped, whereas in comparison, minor cheliped has angular and serrate teeth (Fig. 1; Table S1). In addition, major cheliped, either left or right, showed consistent superiority in size in all three morphometric characters (PL, PD, and PW), except for four right-handed individuals with larger left PW and one left-handed individual with larger right PW. No individual was found to have both large chelipeds.

**Table 1:** The average, maximum, and minimum values of the measured morphometric variables of *Scylla olivacea*.

Variables	Immature male (*n* = 11)	Immature female (*n* = 9)	Mature male (*n* = 91)	Mature female (*n* = 39)
**BW, g**				
Minimum	72.20	59.60	116.10	102.00
Maximum	118.30	117.10	436.60	301.5
Average	102.95 ± 14.66	91.01 ± 21.21	216.17 ± 72.27	206.65 ± 42.76
**CW, mm**				
Minimum	75.75	72.18	83.85	83.32
Maximum	88.00	87.18	123.06	125.16
Average	81.29 ± 3.51	80.54 ± 6.09	98.26 ± 8.91	106.42 ± 7.88
**ICW, mm**				
Minimum	72.57	68.73	63.17	78.08
Maximum	85.18	89.36	179.72	119.78
Average	77.83 ± 3.77	77.69 ± 7.11	94.47 ± 9.36	101.94 ± 7.55
**CL, mm**				
Minimum	50.91	47.64	55.69	54.29
Maximum	59.48	60.55	96.8	86.61
Average	54.74 ± 2.85	54.35 ± 4.84	66.63 ± 6.83	71.44 ± 5.59
RDL, mm				
Minimum	22.36	20.39	20.63	25.31
Maximum	27.17	29.68	56.58	38.41
Average	25.00 ± 1.65	24.26 ± 3.04	33.99 ± 6.21	32.14 ± 3.16
RPL, mm				
Minimum	49.48	45.72	49.36	51.27
Maximum	61.35	57.83	98.50	80.89
Average	54.69 ± 3.54	51.51 ± 4.20	72.21 ± 10.61	65.23 ± 6.39
RML, mm				
Minimum	26.46	24.19	30.32	30.28
Maximum	39.29	34.00	67.94	53.64
Average	34.01 ± 3.67	28.83 ± 3.56	41.39 ± 5.84	36.64 ± 4.77
RPD, mm				
Minimum	17.47	16.78	16.78	19.30
Maximum	25.48	22.25	47.43	34.23
Average	20.36 ± 2.18	19.14 ± 2.13	30.60 ± 7.01	25.54 ± 3.67
RPW, mm				
Minimum	11.08	11.06	11.06	11.50
Maximum	19.02	19.66	30.99	31.93
Average	13.58 ± 26.04	13.21 ± 2.69	19.62 ± 4.08	16.42 ± 3.35
LDL, mm				
Minimum	22.12	20.43	22.75	22.90
Maximum	30.66	29.73	49.12	39.01
Average	26.04 ± 2.73	24.33 ± 2.94	33.41 ± 5.51	32.56 ± 3.17
LPL, mm				
Minimum	48.47	45.1	51.50	51.73
Maximum	60.85	57.02	96.54	76.64
Average	54.52 ± 3.87	51.15 ± 4.20	70.25 ± 9.58	64.89 ± 4.30
LML, mm				
Minimum	27.05	24.22	31.25	30.88
Maximum	39.80	32.86	55.30	63.89
Average	33.37 ± 3.63	28.53 ± 3.30	40.81 ± 4.89	36.40 ± 5.16
LPD, mm				
Minimum	10.08	15.58	17.38	19.14
Maximum	27.33	20.91	42.54	30.44
Average	19.61 ± 4.20	18.05 ± 1.92	28.97 ± 5.90	24.30 ± 2.50
LPW, mm				
Minimum	9.77	10.68	11.88	11.14
Maximum	19.32	13.45	32.02	18.53
Average	13.60 ± 2.35	12.56 ± 1.14	18.82 ± 3.37	15.52 ± 1.55

**Notes.**

BW body weight CW carapace width ICW internal carapace width CL carapace length RDL right dactyl length RPL right propodus length RML right merus length RPD right propodus depth RPW right propodus width LDL left dactyl length LPL left propodus length LML left merus length LPD left propodus depth LPW left propodus width

**Figure 2:**
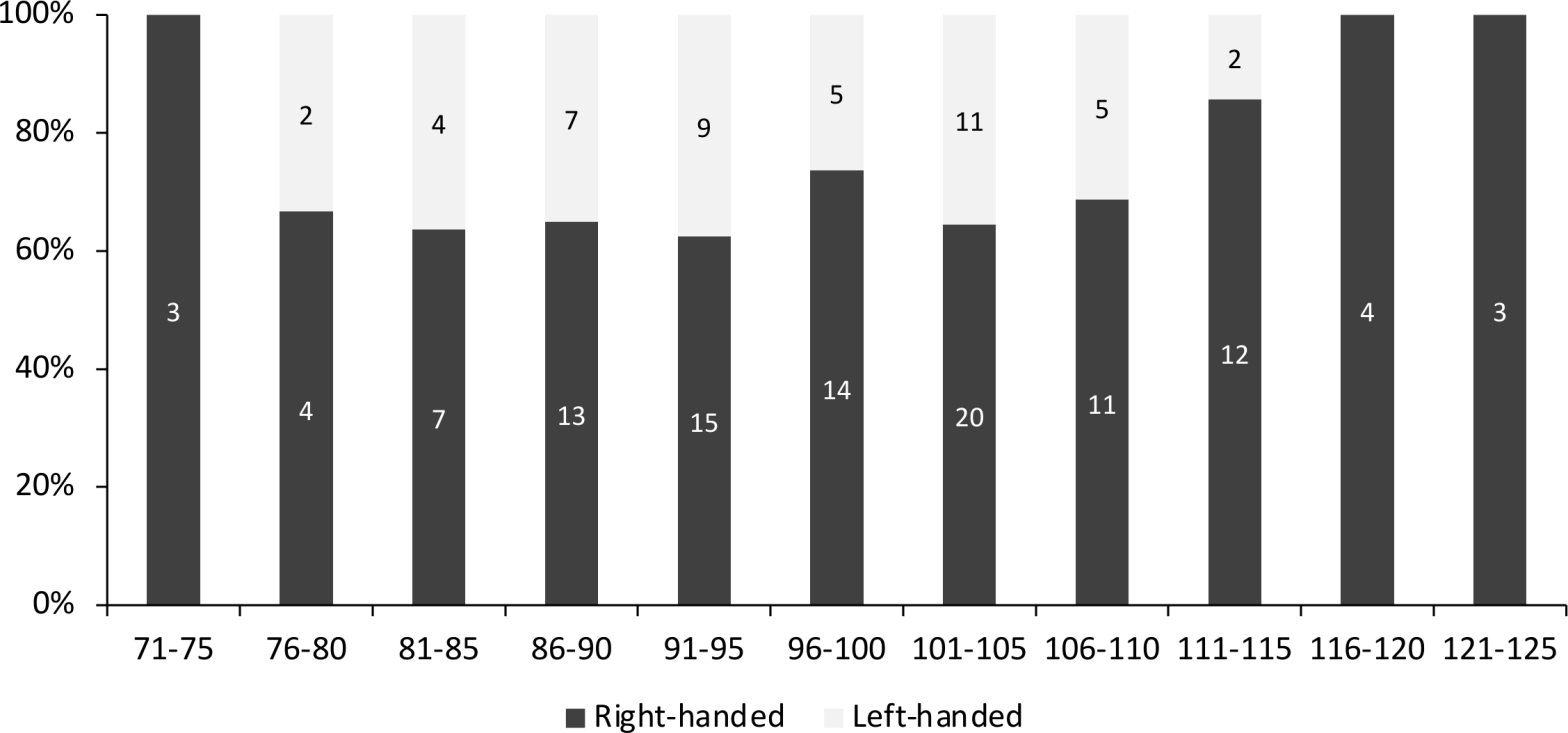
The percentage of right- and left-handed *Scylla olivacea* according to five mm carapace width (CW) size class.

Overall, 70.2% of the measured individuals were right-handed (106 out of 151 individuals). When divided according to size class, left-handed individuals were concentrated between 76-115 mm CW, and the highest percentage of left-handed crabs (37.5%) was found between the size of 91 to 95 mm CW (Fig. 2). There was no significant association between handedness and sex (*χ*^2^(1) = 0.023, *P* = 0.880, Cramer’s *V* = 0.012), between handedness and maturation status (*χ*^2^(1) = 0.0004, *P* = 0.983, Cramer’s *V* = 0.002), and between handedness and CW size class (*χ*^2^(10) = 7.662, *P* = 0.662, Cramer’s *V* = 0.225) of *S. olivacea*. Additionally, the logistic regression model (Hosmer and Lemeshow test; *χ*^2^(8) = 3.303, *P* = 0.914) with a correct classification of 99.1% indicated that all five predictor variables, *i.e.,* sex, maturation status, CW, ICW, and CL, failed to predict handedness status in *S. olivacea* (Table 2).

**Figure 3:**
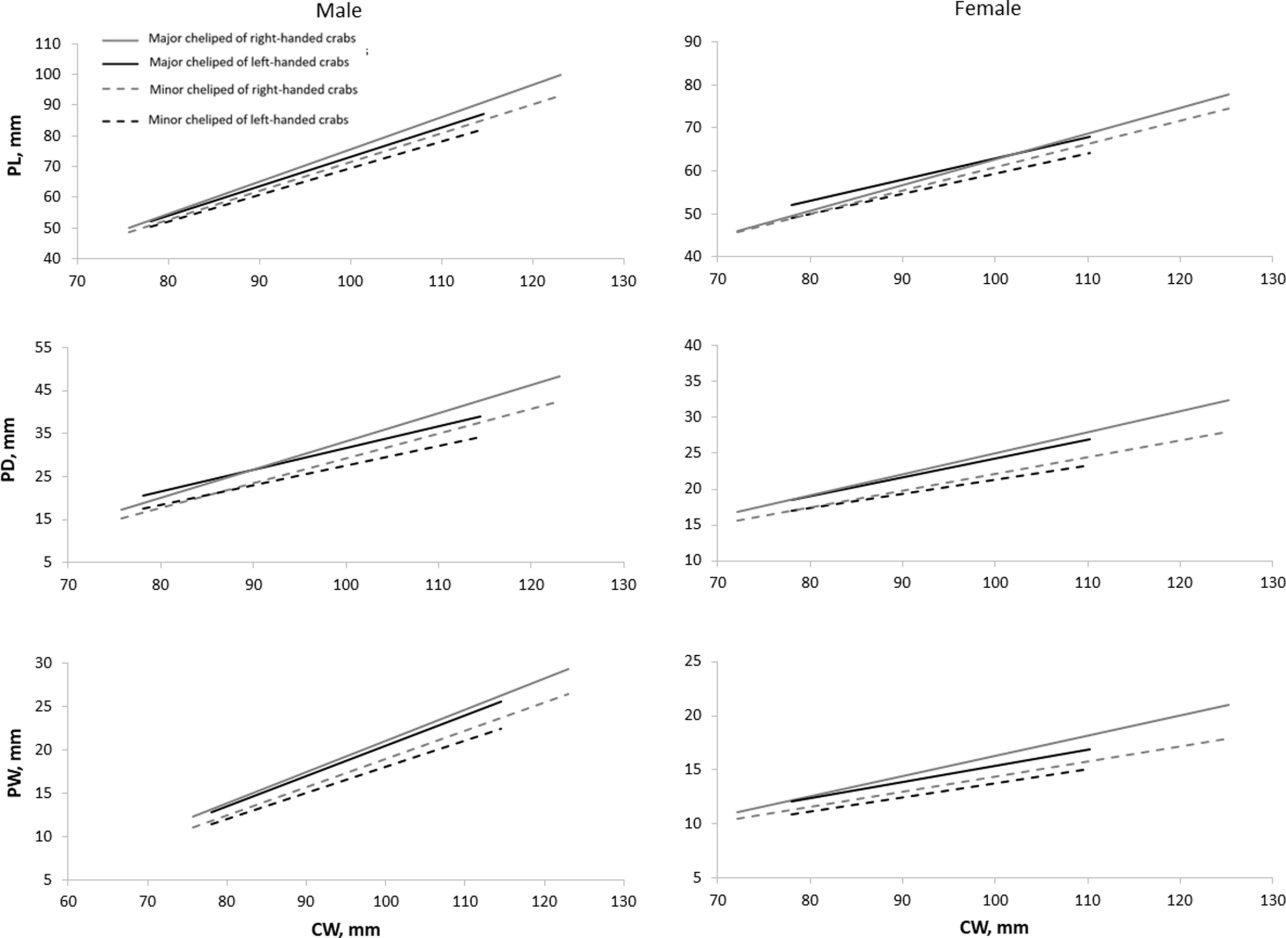
Relationship of carapace width (CW) and cheliped characters (*i.e.,* propodus length (PL), propodus depth (PD), and propodus width (PW)) on major and minor chelipeds of *Scylla olivacea*.

All cheliped variables of right-handed individuals exhibited a stronger relationship with CW compared to that of left-handed individuals, as implied by their higher coefficient of determination (R^2^) values, except for the RPW of right-handed females (Table 3). The size of PL, PD, and PW on the major cheliped, regardless of if the major cheliped is left or right cheliped, was always larger than the corresponding variables on the minor cheliped (Fig. 3). Additionally, the size of PL, PD, and PW on both major and minor chelipeds of right-handed individuals was larger than that of left-handed individuals, and this pattern became clearer with increasing body size (CW). Notably, the PL of males and PD of females showed an inverse relationship (the sizes of PL or PD on major and minor chelipeds of left-handed crabs were larger than that of right-handed crabs) when CW was small but reverted to right-handed biased size difference as the CW increases.

## Discussion

Most species of brachyuran crabs, including those of the family Portunidae, are known to be heterochelous (Abby-Kalio & Warner, 1989; Haefner Jr, 2000; Akin-Oriola, Anetekhai & Olowonirejuaro, 2005; Zainal, 2017). The understanding of heterochely in a species would aid in deciphering the potential function of biased cheliped sizes and provide added information to its life history. Although not as prominent as possessing one large cheliped as in some crab species such as fiddler crabs *Uca* spp. (Yamaguchi & Henmi, 2001; Martins & Masunari, 2013), morphometric comparison of cheliped variables suggests the presence of heterochely in mud crab *S. olivacea*, with a higher percentage of individuals being right-handed. The right-handed biasness among brachyuran crabs is common, especially in predatory brachyuran species, in which the presence of right-handed major cheliped would facilitate easier handling of molluscan preys that are often asymmetric, dextrogerous, and exhibit right-handed shell coiling (Goés & Fransozo, 1998; Mariappan, Balasundaram & Schmitz, 2000; Dietl & Hendricks, 2006). The dominant right-handed individuals observed in *S. olivacea* thus could be driven by its natural feeding habits (Seed & Hughes, 1995) as hard-shelled bivalves are among the primary consumed food of mud crabs of the genus *Scylla* (Viswanathan & Raffi, 2015; Ghazali et al., 2017; Fazhan et al., 2022). Similarly, in other portunids that exhibit right-handedness, such as Gazami crabs *Portunus trituberculatus*, Masunari et al. (2020) noticed that *P. trituberculatus* exclusively used their major (right) cheliped to crack open shells. Based on handedness development during the megalopa stage of *P. trituberculatus*, Masunari et al. (2015) postulate that the earliest durophagous crabs favoured the selection of right-handedness, and this character preference subsequently succeeded to extant species. Similarly, right-handedness in the xanthid crab *Eriphia smithii* conferred greater attack success on the dextral snail *Planaxis sulcatus* (Shigemiya, 2003). Future studies on the handedness characteristics of *S. olivacea* during early development stages will be helpful in validating the right-handed inheritability observed in other portunid species.

**Table 2:** Logistic regression predicting likelihood of handedness in *Scylla olivacea* based on sex, maturation status, carapace width (CW), internal carapace width (ICW), and carapace length (CL).

	*B*	S.E.	Wald	df	*P*
Sex	0.036	0.391	0.008	1	0.927
Maturation status	−0.124	0.572	0.047	1	0.828
CW	−0.023	0.040	0.337	1	0.562
ICW	0.001	0.026	0.001	1	0.977
CL	0.045	0.055	0.653	1	0.419
Constant	−1.410	1.789	0.621	1	0.431

**Notes.**

*B* *B* coefficient S.E. standard error

**Table 3:** Regression equations of cheliped morphometric variables of *Scylla olivacea*. Carapace width (CW) was used as the independent variable.

Dependent cheliped variables	Sex	N	Equation	R^2^(100)
Right-handed individuals				
RPL	M	72	Log RPL = 0.9407 + 0.3991 Log CW	91.33
	F	34	Log RPL = −0.9526 + 0.9455 Log CW	95.26
RPD	M	72	Log RPD = 0.1327 + 0.5895 Log CW	89.24
	F	34	Log RPD = −0.935 + 1.1656 Log CW	94.32
RPW	M	72	Log RPW = 0.1439 + 0.5006 Log CW	80.25
	F	34	Log RPW = −0.876 + 1.0424 Log CW	59.48
LPL	M	72	Log LPL = 0.9805 + 0.3715 Log CW	89.02
	F	34	Log LPL = 0.0231 + 0.8807 Log CW	96.35
LPD	M	72	Log LPD = 0.0366 + 0.6069 Log CW	85.05
	F	34	Log LPD = −0.7656 + 1.0545 Log CW	84.88
LPW	M	72	Log LPW = 0.092 + 0.5036 Log CW	85.52
	F	34	Log LPW = −0.734 + 0.9454 Log CW	85.80
Left-handed individuals				
RPL	M	30	Log RPL = −0.7281 + 1.2847 Log CW	76.02
	F	15	Log RPL = 0.2464 + 0.7635 Log CW	61.59
RPD	M	30	Log RPD = −2.0282 + 1.7324 Log CW	67.83
	F	15	Log RPD = −0.5258 + 0.9273 Log CW	78.94
RPW	M	30	Log RPW = −2.2344 + 1.743 Log CW	63.38
	F	15	Log RPW = −0.7227 + 0.9302 Log CW	61.41
LPL	M	30	Log LPL = −0.8059 + 1.3342 Log CW	75.29
	F	15	Log LPL = 0.0867 + 0.8555 Log CW	93.83
LPD	M	30	Log LPD = −1.891 + 1.693 Log CW	61.37
	F	15	Log LPD = −0.7767 + 1.0805 Log CW	80.49
LPW	M	30	Log LPW = −2.2363 + 1.7708 Log CW	59.75
	F	15	Log LPW = −0.6372 + 0.9119 Log CW	67.48

**Notes.**

M male F female RDL right dactyl length RPL right propodus length RML right merus length RPD right propodus depth RPW right propodus width LDL left dactyl length LPL left propodus length LML left merus length LPD left propodus depth LPW left propodus width

The presence of a large-sized molariform tooth on the major cheliped (or also known as the crusher) in *S. olivacea* might also be an evolutionary adaptation to facilitate better grasping and provide added mechanical force (Freire, Sampedro & González-Gurriarán, 1996; Mariappan, Balasundaram & Schmitz, 2000) during feeding of crustaceans and molluscs of various shapes and sizes. Similar presence of a large tooth on the major cheliped is common in other heterochelous crab species and is often used as an indicator for handedness (Yamaguchi & Tokunaga, 1995; Goés & Fransozo, 1998). *S. olivacea* with two major or minor chelipeds was not observed in this study, as all crabs showed the presence of major molariform teeth in one of its chelipeds. Similar absence of individuals possessing two major chelipeds was reported in other portunids, including *Portunus pelagicus* (Zainal, 2017), *Carcinus maenas* (Abby-Kalio & Warner, 1989), *Liocarcinus deputator* and *Macropipus tuberculatus* (Abello, Pertierra & Reid, 1990). In comparison, individuals with two small chelipeds or two large chelipeds have been reported in brachyurans of other families, such as in fiddler crab *Tubuca arcuata* (Yamaguchi & Henmi, 2001; Vale et al., 2015). Since cheliped morphology, including the presence of molariform teeth, influence the generation of force and link with hardness of diet (Schenk & Wainwright, 2001), behavioural and selection studies on various diet types and cheliped shape and size of *S. olivacea* are warranted in the near future.

The 70.2% right-handed *S. olivacea* found in this study fall within the range of other right-handed crab species as well. For example, a range of 78.0% to 82.5% right-handed individuals was reported in *C. sapidus* (Hamilton, Nishimoto & Halusky, 1976; Smith, 1990), 75.3% right-handedness in *Potamon potamios* (Scalici & Gherardi, 2008), 83.5% right-handed crabs in *C. ornatus* (Haefner Jr, 1990), 80.4% right-handedness in *Eriphia gonagra* (Goés & Fransozo, 1998), and 79% right-handed individuals in *C. maenas* (Abby-Kalio & Warner, 1989). Right-handed individuals were found to be dominant in all size classes of *S. olivacea*, with 100% right-handed individuals being observed in the smallest and largest size classes. In other crabs and shrimp species, the reversal of handedness is well documented, such as in crabs *Menippe mercenaria* (Simonson, 1985), *P. trituberculatus* (Masunari et al., 2020), *Callinectes sapidus*, *C. maenas*, *Callinectes ornatus*, *Monomia argentata* (Yamaguchi & Tokunaga, 1995), and snapping shrimp *Alpheus angulosus* (Cooney, Korey & Hughes, 2017). Left-handed crabs risen from the loss of the right major cheliped might not attain the same shape and crushing force as the original right major cheliped (Masunari et al., 2015). However, there are species, such as lithodid crab *Lopholithodes foraminatus* (Duguid, 2010), that will not reverse the direction of cheliped asymmetry despite experiencing limb loss of the major cheliped. Although the event of handedness reversal is yet to be documented in *S. olivacea*, the presence of left-handed individuals in a lesser percentage and only in the sub-adult size classes might suggest that they were originally right-handed crabs and experienced handedness transition due to the chela loss. Mud crabs of the genus *Scylla* are known for their aggressiveness and cannibalistic nature (Fujaya et al., 2020; Kawamura et al., 2020). Sub-adults might have a higher chance of losing their major cheliped during fighting with other larger-sized crabs over food, territory, or mate (Fazhan et al., 2017b) and thus resulting in the occurrence of left-handed individuals at these size classes. In comparison, some species (*e.g.*, *Charybdis bimaculata*) are suggested to innately develop left-handedness based on the similar percentage of left-handedness across size groups (Yamaguchi & Tokunaga, 1995). The increase of left-handed individuals as size increases in *S. olivacea* is also evident in other portunid species (Hamilton, Nishimoto & Halusky, 1976; Haefner Jr, 1990; Norman & Jones, 1991; Smith & Hines, 1991; Yamaguchi & Tokunaga, 1995).

The presence of heterochely in both sexes and the non-association between handedness and sex, as observed in males and females of *S. olivacea,* is commonly observed in other crab species as well (Abello, Pertierra & Reid, 1990; Goés & Fransozo, 1998; Scalici & Gherardi, 2008). We postulate that the similar heterochely phenomenon in both sexes of *S. olivacea* might be related to the shared biological functions of having a major cheliped, such as in food acquisition (Scalici & Gherardi, 2008) and territorial guarding (Mariappan, Balasundaram & Schmitz, 2000). Interestingly, major and minor chelipeds of right-handed individuals were larger compared to that of left-handed individuals. One of the possible explanations is that handedness reversal in crabs that have lost their right major cheliped might take up to several moults (Hamilton, Nishimoto & Halusky, 1976; Simonson, 1985). Left-handedness in a predominantly right-handed population has been related to lower aggressiveness (less likely to initiate a fight, engaged in less fights, and less likely to win a fight) in crabs such as *Uca* spp. (Backwell et al., 2007). Handedness reversal is still undocumented in *Scylla* spp., and its validation in the future experiment could provide the basis for the optimisation of mud crab culture *via* the development of a less aggressive left-handed dominant mud crab population.

## Conclusions

In conclusion, the knowledge that *S. olivacea* exhibits heterochely and is predominantly right-handed provides a basis for understanding its ecological functions. In addition to traditional morphometric indices to discern heterochely, future studies could look into new indices –heterometry (ratio between the ‘size index’ of chelipeds) and heteromorphy (numerical quantification of shape differences between chelipeds) (Spani et al., 2020), and correlate them with ecological indices. As we show that heterochely and handedness occur in mud crabs, future aquaculture practices might benefit by exploring the potential lesser impact of autotomy of its minor instead of major chelipeds during moult induction practices during soft-shell crab production (Waiho et al., 2021).

##  Supplemental Information

10.7717/peerj.15143/supp-1 Table S1 Morphometric measurements of mud crabs Scylla olivacea

## Additional Information and Declarations

### Data Availability

The following information was supplied regarding data availability: The raw measurements are available in the Supplementary File.
